# Effects of training on plasmatic cortisol and testosterone in football female referees

**DOI:** 10.14814/phy2.15291

**Published:** 2022-05-04

**Authors:** Antonella Muscella, Giulia My, Selmi Okba, Daniele Zangla, Antonino Bianco, Santo Marsigliante

**Affiliations:** ^1^ Department of Biological and Environmental Science and Technologies (Di.S.Te.B.A.) University of Salento Lecce Italy; ^2^ Higher Institute of Sports and Physical Education of Kef University of Jendouba Jendouba Tunisia; ^3^ Sport and Exercise Sciences Research Unit Department of Psychology, Educational Science and Human Movement University of Palermo Palermo Italy

**Keywords:** cortisol, football, maximal oxygen consumption (VO2max), testosterone, training, Yo–Yo intermittent recovery test level 1 (YYIRT1)

## Abstract

There is very little about the impact that sports training has on female football referees. Therefore, we determined the effects of a 40‐week physical preparation, including a full football season, on plasma testosterone and cortisol concentrations and physical performance in female football referees. Plasma cortisol and testosterone concentrations were assayed together with fitness tests at the beginning of the training period (T0, in September), after 8 weeks from T0 (T1), at the mid of the season (T2, 24 weeks after T0), and at the end of the season (T3, in June, 40 weeks after T0). Plasma cortisol increased during the first period and up to T2 (from 15.4 ± 4.7 to 28.5 ± 3.9 µg/dl; *p* < 0.001), and then decreased at the end of the season (T3: 16.0 ± 2.4 µg/dl). Plasma testosterone concentration in T0 was 14.2±0.37 µg/dl and increased in T1 (57.1 ± 3.7 µg/dl) and T2 (47 ± 3.7 µg/dl) and then decreased in T3 (33.5 ± 2.8 µg/dl). Resting testosterone levels in women were very low (14,2 ± 0.37 µg/dl) (Figure 3c). Testosterone increased in T1 (57.1 ± 3.7 µg/dl) and T2 (47 ± 3.7 µg/dl) whilst, at the end of the season, its concentration decreased (33.5 ± 2.8 µg/dl) (Figure 3c). Significant improvements were observed in all physical performances during the observed period (ANOVA, *p *< 0.05). Finally, testosterone and cortisol concentrations significantly (*p *< 0.0001 for both) correlated with maximal oxygen consumption. In T1, testosterone concentration was also significantly correlated with running speed test (*p *< 0.001). In conclusion, training induces endocrine changes in order to maintain body homeostasis in women referees. It is important that coaches and sports scientists regularly observe changes in endocrine function induced by training and matches in female referees, because they can help maximize referees’ performance and limit cases of overtraining.

## Graphical abstract

Since biological sex can influence the physical performance and the hormonal response to exercise, plasma cortisol and testosterone concentrations were assayed together with fitness tests before the training period (T0), immediately after T0 (T1), at the middle (T2) and at the end (T4) of the season, in 14 female referees.Significant improvements were observed in all physical performances during the observed period. Cortisol and testosterone changes significantly correlated with maximal oxygen consumption. In T1, testosterone concentration was also significantly correlated with running speed test.

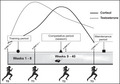

## 1 INTRODUCTION

Football, the most popular and currently most played sport in the world (Keen, 2018) has more and more interested researchers who study this complex sport in its many aspects. Noteworthy, most of the research has only studied the performance of football players (Djaoui et al., 2017; Fernandes‐da‐Silva et al., 2016; Hill‐Haas et al., 2011; Mello et al., 2017; Montini et al., 2017; Muscella et al., 2019; Slimani et al., 2017; Stolen et al., 2005). Match referees are indispensable, independently of competition level or age classes. As it is known, competitive football matches are regulated by a referee together with two assistant referees and a side‐line official that can be officials of either sex, provided they possess a high standard skill (fourth official) (FIFA. Laws of the Game 2013/2014.2012). Since football matches frequently have low scores (about 2.7 goals per game), the referees have significant implications on the outcome of the matches (Abt & Barry, 2015). As a result, referees must pay great attention to footballers’ movements also to prevent injuries and avoid violating the game rules. For these reasons football referees must be physically well trained, at the level of football players, to keep up and to reach optimal positioning when making critical decisions (Mallo et al., 2012). Nevertheless, there are few studies, focusing on football referees and assistants (Barbero‐Alvarez et al., 2012; Bizzini et al., 2012; Castillo et al., 2016, 2017; Krustrup & Bangsbo, 2001). Referees’ high‐speed running distances decline during the later stages of the match (Krustrup & Bangsbo, 2001) perhaps a sign of accumulated fatigue (Krustrup et al., 2009) due to the high physical and physiological match demands (Mallo et al., 2012), as demonstrated by blood lactate increment and in sprint performance decrement (Castillo et al., 2016). Nowadays, the number of football matches is very increased and consequently the referees have additional fatigue during the competitive season (from Silva et al., 2011). So, making the football referee is an important physical challenge even because referees are almost always older than footballers (Weston et al., 2010).

While male and female athletes are roughly numerically equal, the referees are mostly male at a professional, local and national level. Nevertheless, football referees of both sexes train for the same time during pre‐ season (7.5 ± 3.1 vs 7.1 ± 3.4 h/week, female vs male) and in‐ season (6.0 ± 2.9 vs 6.1 ± 2.4 h/week, female vs male) (Bizzini et al., 2012; Lima e Silva et al., 2019). In the only study addressing the match performances of female referees, a total distance of 9.6–10.5 km was covered, with only 1300 m performed at high intensity that represented 13% of match's total distance compared with the corresponding 26–58% observed in male referees (Castagna et al., 2007) was reported (Mallo et al., 2012). Therefore, the football referees of both sexes undergo to physical and psychological stress, because of the complex decision‐making process when facing players, coaches and public. Physical training concerns the state of the hormones of the hypothalamic–pituitary–gonadal axis hormones in both men and women (Hackney et al., 2003); this is especially true for high intensity and physical challenging sports (Aldous et al., 2016; Hammami et al., 2017). The glucocorticoid hormone cortisol plays a prominent part in the regulation of physical and mental stress. Most catabolic adaptations to exercise are regulated by cortisol, with its circulating concentration depending upon exercise intensity and duration and physical fitness level (Dickerson & Kemeny, 2004). Testosterone is a steroid hormone abundantly secreted from the testes and less by adrenal cortex and ovaries. Testosterone has an important role in muscle hypertrophy, and it enhance athletic performance in men and women (Wood & Stanton, 2012). Thus, testosterone and cortisol values are useful in assessing the impact of competition and training as a reflection of the anabolic and catabolic processes balance (Urhausen et al., 1995). Notwithstanding some studies examining seasonal physical performance adaptation of referees (Weston et al., 2004, 2011), no study has analyzed the impact of the above‐mentioned mental and physical stress on testosterone or cortisol over a long time with particular concern to female referees.

Very recently we have evaluated the effects on cortisol and testosterone and physical performance of an entire football season in male referees, showing that training leads to testosterone and cortisol variations to promote physiological adaptations (Muscella et al., 2021). In the present paper we repeated the same study on female referees in order to analyze whether there are significant differences in adaptive responses to athletic training or if there are no gender‐related differences. Thus, we have analyzed the effects of a whole football season period on female football referee's physical performance and on blood cortisol and testosterone levels using the same training protocol as in the previous study on male football referees (Muscella et al., 2021).

## 2 METHODS

### 2.1 Procedures

**FIGURE 1:**
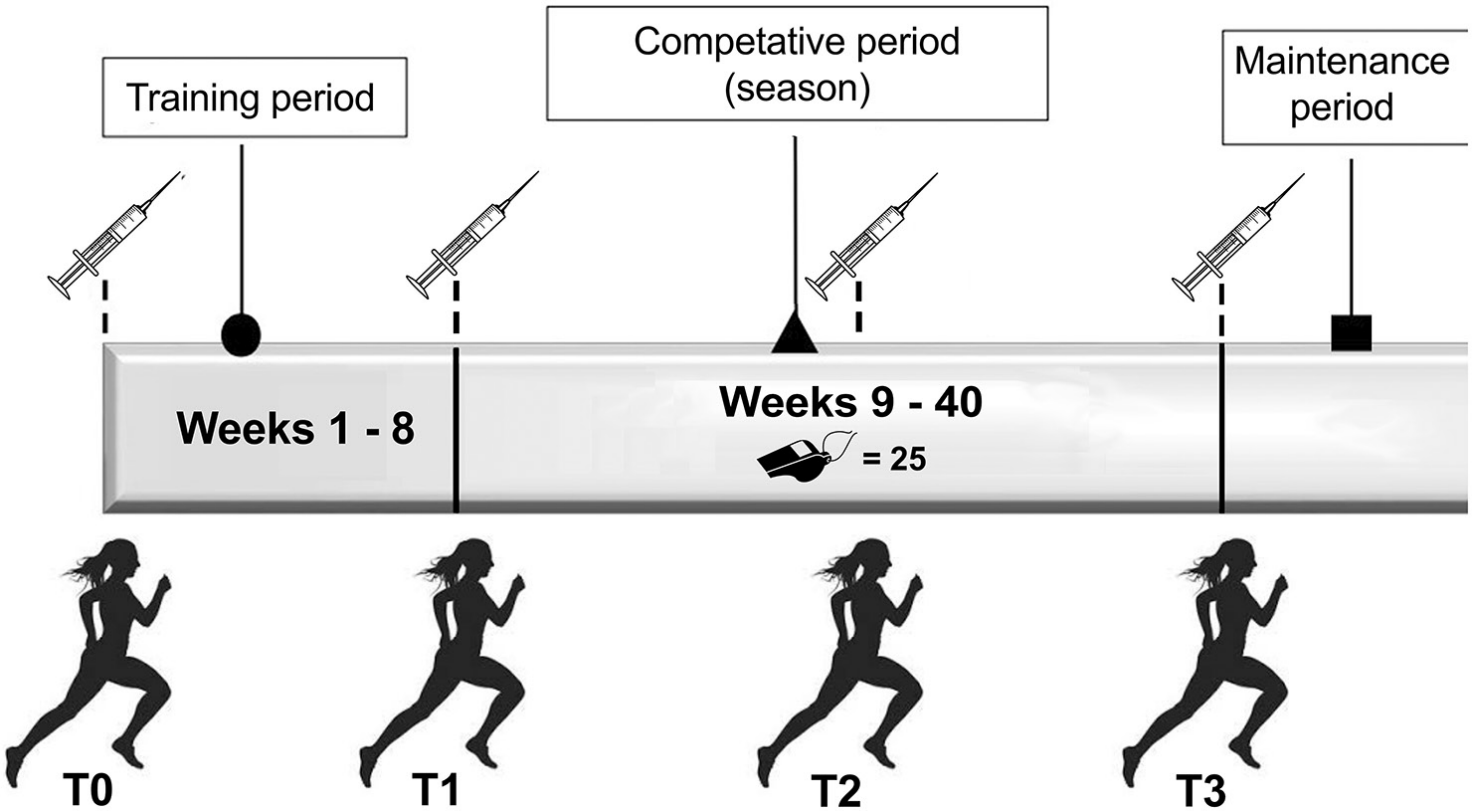
Organization of the research study protocol applied during a football season (40 weeks). Testing time‐points were four (indicated by the stars): before the beginning of the training period (T0, in September, week 0), just after the training period (T1, 8 weeks from T0), at the middle of the season (T2, 24 weeks after T0), and at the end of the season (T3, 40 weeks after T0). The number of match's officiated (25) is shown next to the whistle logo

This study analyzed the effects of a 40‐week physical preparation period on cortisol and testosterone blood concentrations and physical performance in football female referees. The results of fitness tests evaluating linear sprint, change of direction and intermittent high‐intensity performance in football referees during the competitive season 2018/2019, were also assessed. Fitness and biological tests were performed at beginning of the training period (T0, in September), after 8 weeks from T0 (T1), at the mid of the season (T2, 24 weeks after T0), and at the end of the season (T3, in June, 40 weeks after T0), as reported in Figure 1.

The blood samples were collected at 7:30 am, in the fasting state.

According the 2016 FIFA Fitness Tests document, the referees who participated to study, were refereeing at category 1 (Referees who officiate in the professional competitions organized by a professional league) and category 2 (Referees who officiate in the semi‐professional and amateur competitions organized at national level).

### 2.2 Participants

This study was organized as a previous study carried out on male football referees (Muscella et al., 2021). Briefly: twenty‐four females football referees that officiated official football matches during the 2017/2018 season volunteered to participate in this study. The age of referees was in the range 18–22 years. According the 2016 FIFA Fitness Tests document, the referees who participated to this study, were refereeing at category 1 (Referees who officiate in the professional competitions organized by a professional league) and category 2 (Referees who officiate in the semi‐professional and amateur competitions organized at national level). A control group composed by 24 non‐athletic women of the same ages (range 18–23 years), was recruited volunteered. The control group did not receive any specific exercise protocol and were allowed to continue their training routine (4 ± 2 sessions per week). During the study, subjects were instructed to maintain their normal training diet and ingest only water in the 60 min before data collection. None of them smoked or had a significant medical or health history. Both referees and non‐athletic women took no medication including oral contraceptives before or during this study, nor were they taking any supplements or corticoids.

The study was approved by Institutional Review Board (I.R.B.) of Department of Biological and Environmental Science and Technologies (Di.S. Te.B.A., N.1 /2021), and all experiments were conducted in accordance with the 2013 Helsinki declaration and its later amendments. Written informed consent was obtained from each participant after full explanation of the purpose and nature of all procedures used.

### 2.3 Anthropometric characteristics

Body weight and height were obtained with standard techniques as previously reported (Jackson & Pollock, 1978). Resting Heart Rate (HR) was measured by theolar S710 heart rate monitor and data were processed by specific software. All measurements were taken at 7.30 a.m. by the same investigator, for all time periods. Anthropometric characteristics of referees, determined before the beginning of the training period (mean ± SD), are shown in Table 1.

**TABLE 1:** Physical and fitness characteristics of the study participants

Physical characteristic	Referees		Control subjects	
	Pre	Post	Pre	Post
Age (years)	19,7 ± 0.6	20,5 ± 0.5	18,9 ± 0.6	20,1 ± 0.8
Height (cm)	168 ± 7.3	168 ± 7.3	167 ± 6.5	167 ± 6.5
Weight (kg)	62.6 ± 5.8	59.3 ± 4.2*	64.2 ± 7.2	64.6 ± 8.7**
BMI (Kg m^−2^)	21.7 ± 0.6	21.1 ± 0.7	22.3 ± 0.6	23.2 ± 0.4**
%Body fat	23.9 ± 3,8	21.7 ± 4.6*	24.5 ± 4.5	25.1 ± 3.6**
VO_2max_ (ml·Kg^−1^ min^−1^)	49.9 ± 1.7	52.3 ± 1.2*	42.7 ± 3.9**	42.8 ± 3.7**

Note

Data are presented as means (±SD).

* *p *< 0.05 Significantly different between pre and post season

** *p *< 0.05 Significantly different from control subjects.

### 2.4 Training programs

All football referees, when healthy, attended a training program as previously reported (Muscella et al., 2021). The program consisted at least 3 training sessions per week; during the season they performed: 85 sessions of aerobic‐type training (resistance), 65 sessions of anaerobic alactacid type training (speed), 93 sessions of anaerobic lactacid type training (resistance to the speed) and 40 sessions for the improvement of the muscular strength. All referees were regularly trained 120–150 min per session, one session per day, and played on average one competitive match per week. Thus, football referees have been involved in officiating 25 matches during the year. Physical loads during training sessions and the match were quantified using a global positioning sensor (GPS) watch (Timex Ironman Global Training, USA).

During training, football referees reach average heart rate values (HR) of the maximum heart rate (HR_max_) of 164 beats/min that correspond to approximately 85–90% of the maximum heart. On some occasions they reach 97% of their HR_max_.

Training hours per week during pre‐season (7.6 ± 3.1 h/week) and in‐season (6.3 ± 2.9 h/week). Each training session consisted of a 10 min warm‐up followed by either long‐duration running intervals (4 × 4 min or 8 × 2 min) or short duration running intervals (16 × 1 min or 24 × 30 s) with a 2:1 ratio between exercise and rest. Since nutritional guidelines were provided to all referees, they followed the same nutritional and hydration protocol during the period considered (40 weeks).

### 2.5 Physical fitness characteristics

To assess the physiological state of the football referees we gave the tests that have been normally used as part of their match selection criteria: Yo–Yo intermittent recovery test level 1 (YYIRT1) and running speed test. The YYIRT1 was used as a predictor of high‐intensity aerobic capacity and VO_2max_ (Hammami et al., 2017; Krustrup & Bangsbo, 2001; Krustrup et al., 2003). Each participant continues running between two parallel lines 20 meters apart, at a progressively increasing speeds controlled by the “beeps” on a CD. The subjects had a 10 sec active rest period (decelerating and walking back to the starting line) between each running bout. YYIRTL1 was also used to estimate VO_2max_ (ml min^−1^ Kg^−1^), by the equation of Bangsbo (Krustrup & Bangsbo, 2001). In the running speed test, the participants performed three maximal 40 m sprints, measured with an infrared photoelectric cell (Speed‐ trap II Wireless Timing Sistema; Brower Timing System, Draper, UT), as previously described (Muscella et al., 2019).

### 2.6 Blood analysis

Blood samples were taken at the same time of day as previously reported (Muscella et al., 2019).

### 2.7 Statistic analysis

Data, collected in a blinded fashion, were analyzed by GRAPHPAD PRISM 5 software (GraphPad Software, La Jolla, CA, USA). All variables used in the study were checked for normality of distribution before the analyses (Kolmogorov‐Smirnov tests). Results are expressed as means ± standard deviations (SD). Significant differences between time points were assessed by ANOVA followed by the Bonferroni test. Significant differences between T0–T1, T1–T2 and T2–T3, and T0–T3 were evaluated by *t*‐test. The statistical potential associations between changes in cortisol and testosterone with physical parameters (40 m, YYIRTL1 and VO_2max_) were tested using Spearman correlation coefficient (r). *p* < 0.05 was accepted as a level of statistical significance.

## 3 RESULTS

### 3.1 Anthropometric characteristics

Table 1 shows the anthropometric characteristics (height, body mass index, % body fat and weight) of female football referees and control subjects measured before (Pre) the beginning and at the end (Post) of the season (mean ± SD).

### 3.2 Physical fitness characteristics

**FIGURE 2:**
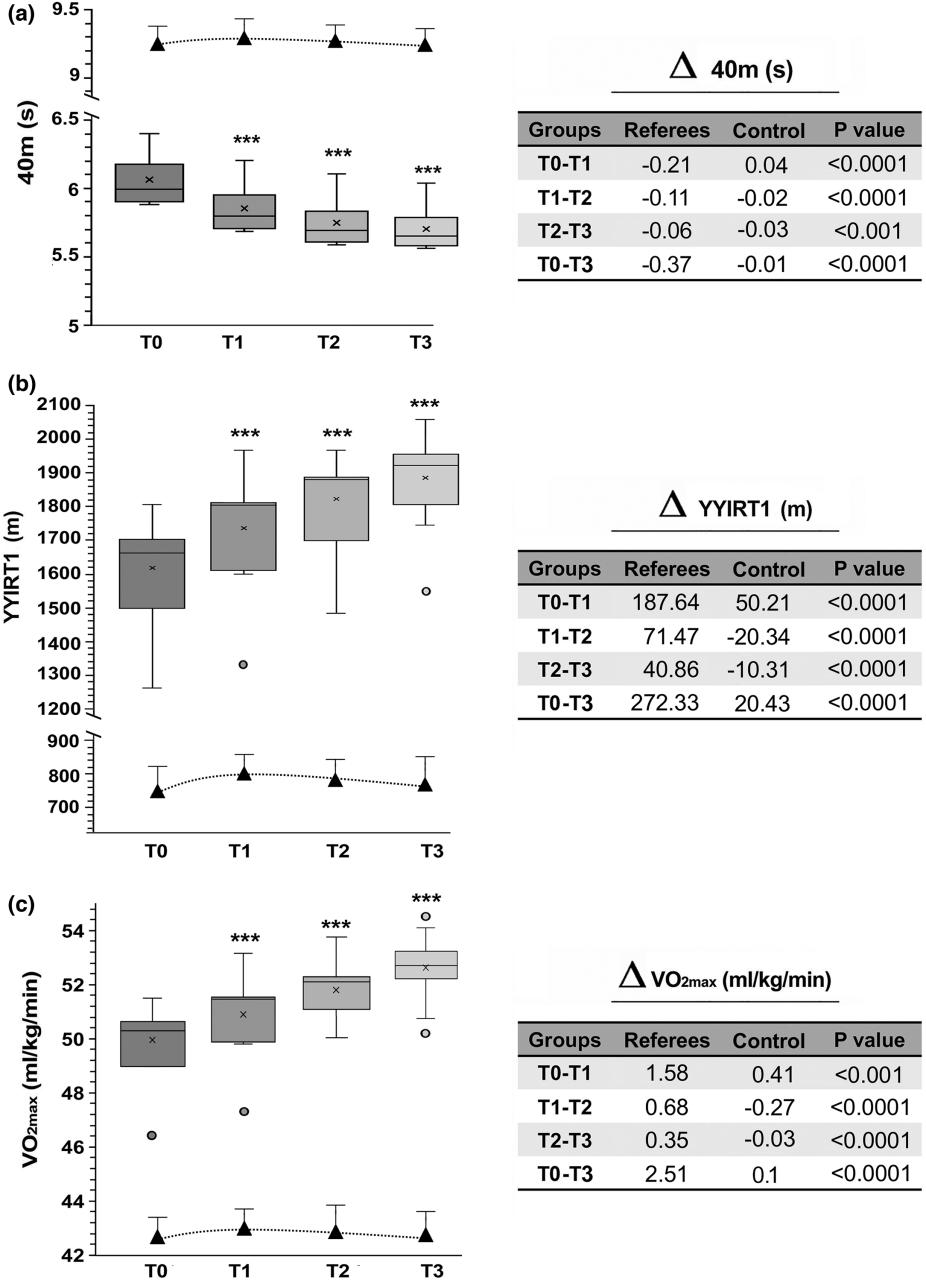
Physical parameters in female football referees and controls subjects and their evolutions during a football season follow‐up. Running speed test (a), Yo–Yo intermittent recovery test level 1 (YYIRT1, b), and maximal oxygen consumption (VO_2max_, c). The football referees were evaluated at four different time points (before the beginning of the training period T0, just after the training period T1, at the middle of the season T2, and at the end of the season T3). In this representation, the central box covers the middle 50% of the data values, between the upper and lower quartiles. The bars extend out to the extremes, while the central line is at the median. Those values which are beyond 1.5 times the interquartile range beyond the central box are plotted as individual points. Significant differences between groups were evaluated by ANOVA test. The mean ± SD values of the control group evaluated at the four different time points are shown with black triangles joined by a dashed line. The differences (Δ) of running speed tests, YYIRT1 and VO2max, between each time point, during a football season follow‐up are shown in the tables. Significant differences between time points were assessed by ANOVA followed by the Bonferroni test. Significant differences between T0–T1, T1–T2 and T2–T3, and T0–T3 were evaluated by *t*‐test

The changes in physical parameters: VO_2max_ (ml min^−1^ Kg^−1^), Yo‐Yo intermittent recovery tests (YYIRT1, m) and 40 m Sprint test (s) were evaluated at four different time points: before the beginning of the training period (T0), at the start of the pre‐season (T1), at the middle of the season (T2), and at the end of the season (T3). Significant increases were observed in the physical parameters throughout the observed period (40 weeks; ANOVA, *p* < 0.05) (Figure 2).

Before the beginning of the training period, sprint times for the 40 m was 6.05 ± 0.20 s (Figure 2a).

In T0, YYIRT1 showed a mean of 1615.7 ± 156 m (Figure 2b); the mean distance covered by the referees during the YYIRT1 increased significantly from T0 to T3, from T0 to T1, and T1 to T2 (Figure 2c and d). Significant differences (*p *< 0.05) were also noted for VO_2max_ in T0‐T1, T1‐T2 and in T2‐T3, (Figure 2c).

### 3.3 Hormonal Measures

**FIGURE 3:**
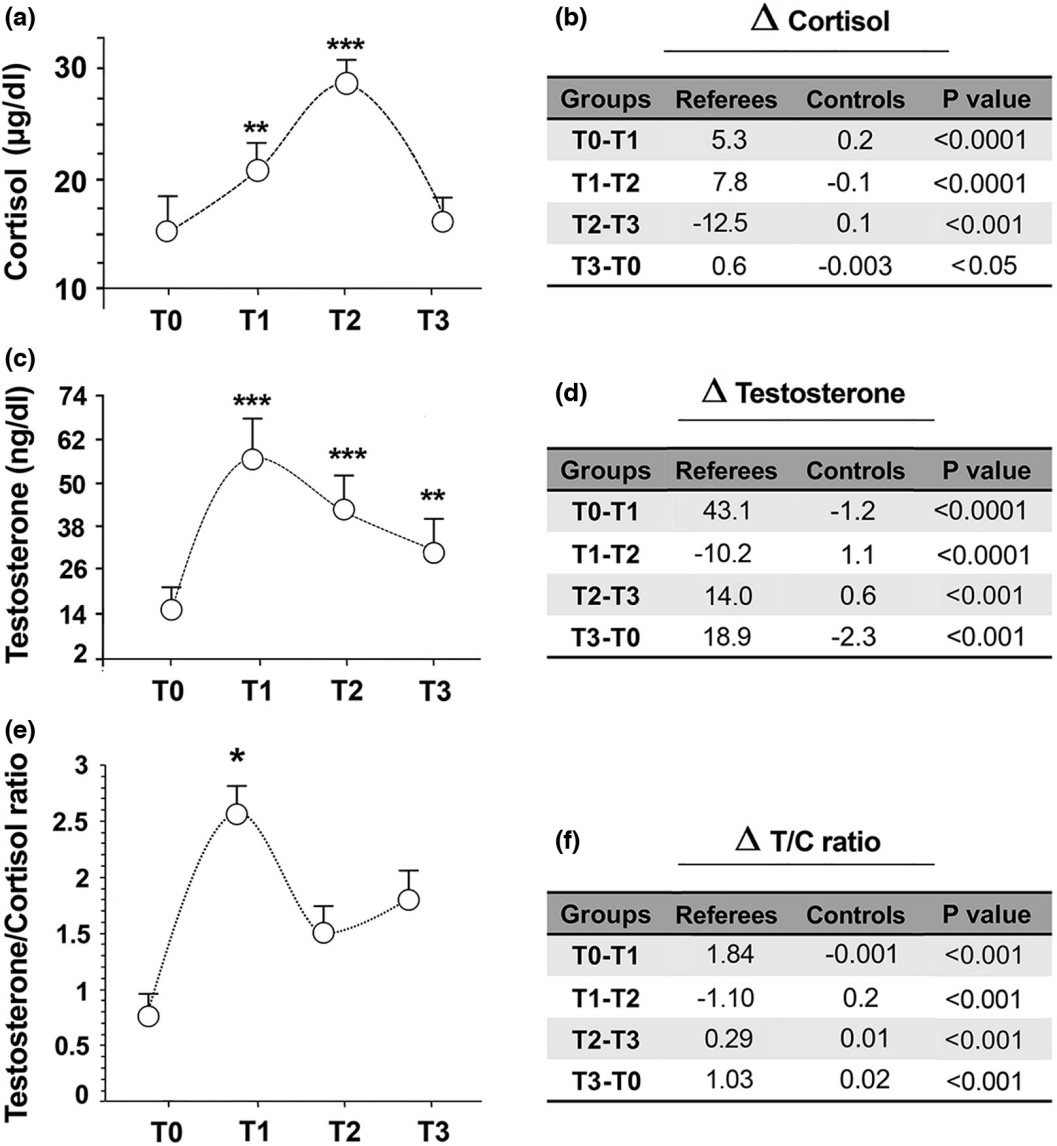
The effects of training on serum cortisol and testosterone in female football referees. Blood cortisol (a) and testosterone (c) concentrations were analyzed at each point‐time during the football seasons follow‐up (mean ± SD). *p < 0.05; **p < 0.01; ***p < 0.001 by ANOVA followed by Bonferroni test between each point‐time and T0. (e) Testosterone to cortisol ratios were analyzed each point‐time during the football seasons follow‐up (mean ± SD). (b, d, f) Tables show the differences in concentrations of cortisol (b) and testosterone (d), and testosterone to cortisol ratios (f) between each point‐time in football referees compared with control subjects. *p* values obtained by *t*‐test show differences in Δ values between each point‐time in referees compared with control subjects

Before the beginning of the training period (T0), no significant differences were noted between the cortisol concentrations in referees and non‐athletic women (data not shown). In referees, cortisol concentrations increased at the first training period (from 15.4 ± 4.7 µg/dl to 20.7 ± 6.1µg/dl *p* < 0.01); then it strongly further increased during mid‐season (T2: 28.5 ± 3.9 µg/dl, *p *< 0.001) and decreased at the end of the season (T3: 16.0 ± 2.4 µg/dl) (Figure 3a).

Resting testosterone levels in women were very low (14,2 ± 0.37 µg/dl) (Figure 3c). Testosterone increased in T1 (57.1 ± 3.7 µg/dl) and T2 (47 ± 3.7 µg/dl) whilst, at the end of the season, its concentration decreased (33.5 ± 2.8 µg/dl) (Figure 3c).

None of the variations in hormonal concentrations were regarded clinically relevant to a player's health since it all rested within the normal range, as documented by a number of other studies (Hammami et al., 2017; Hansen et al., 1999; Makras et al., 2005).

The T/C ratio has been used as a performance index for athletes (Meeusen et al., 2013).

The T/C ratio significantly increased in T3 and in T1 in referee group (<0.05) (Figure 3e). A T/C decrement of more than 30% suggests a state of overtraining (Roli et al., 2018) which, in our study, did not occur with respect to the starting period T0 (Figure 3e).

As shown in Figure 3b and d, serum testosterone and cortisol concentrations were also analyzed in control subjects and no significant differences were found between each point‐time. Significant differences were found in cortisol and testosterone concentrations and testosterone to cortisol ratios between each point‐time in football referees compared to control subjects (Figure 3f).

### 3.4 Correlations between hormone concentrations and physical performances in football referees

**FIGURE 4:**
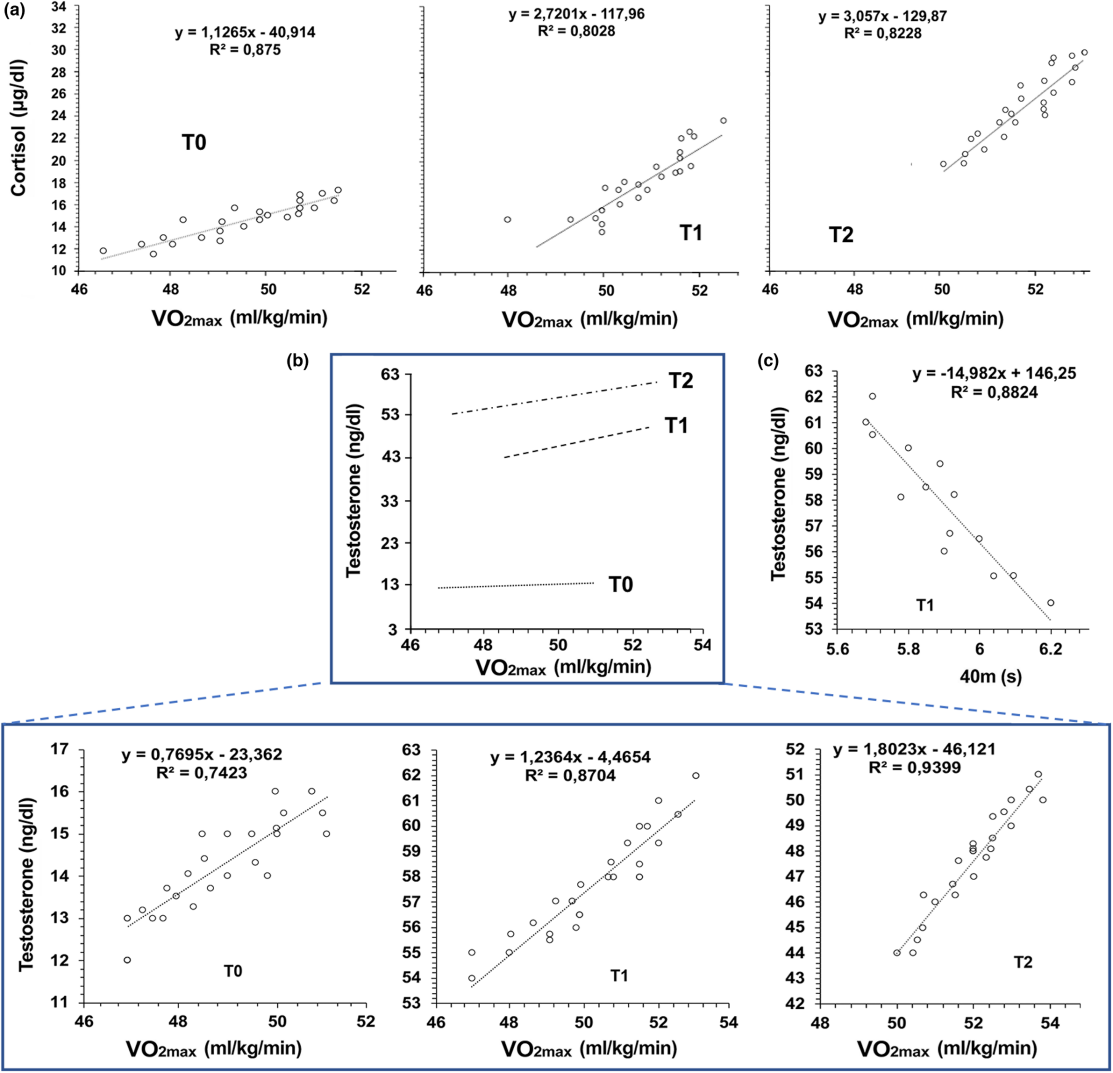
Correlations between testosterone and cortisol variations and physical performance. (a) Scatter plots of cortisol concentration values and VO_2max_ with linear regression at each point‐time during the football seasons’ follow‐up. *p*‐values obtained by Spearman's rank correlation were *p* < 0.0001. (b) Scatter plots of testosterone concentration values and VO_2max_ with linear regression at each point‐time during the football seasons’ follow‐up. (up) values are represented using the same testosterone concentration scale; (down) the testosterone values are shown using different scales at each point‐time. (c) Scatter plots of testosterone concentration values and 40m with linear regressions at the end of the training period (T1). *p*‐values obtained by Spearman's rank correlation were *p* < 0.0001

Both testosterone and cortisol serum concentrations significantly correlated to VO_2max_ (*p *< 0.0001 for all by Spearman's rank correlation, Figure 4a, b) at each point‐time during the football seasons’ follow‐up. After the training period (T1) plasma testosterone concentrations only correlated with the running speed test (*p *< 0.0001 by Spearman's rank correlation, Figure 4c).

As these correlations were not seen in control subjects (data not shown), the exercise intensity affected the response of the hypothalamic‐pituitary adrenal axis. As a whole, these findings sustain the view that moderate to high intensity exercise increases serum testosterone and cortisol concentrations.

## 4 DISCUSSION

Physical activity strongly stimulates the endocrine system; the hormonal response to exercise is controlled by many factors, comprehending individual level of training, mode of exercise, duration, intensity, and the training status of the subject (Karkoulias et al., 2008). Most of the studies, performed at the beginning and at the end of sports performance (pre and post), have evaluated the relationship between exercise and the endocrine system (França et al., 2006; Oliveira et al., 2009; Tremblay et al., 2005). Few studies have analyzed the impact of many months of physical activity on hormonal changes and, unexpectedly, there has been no study that has attempted to comprehend the result of exercise duration on blood hormone concentrations in female football referees. Nonetheless, it is well‐known that competitive, continuous and regular sport exercise affects endocrine homeostasis by specific change of circulating testosterone and cortisol concentrations. Biological sex can influence the physical performance; for example, in sport dependent to strength and endurance, male athletes are 10–15% advantaged than female athletes. This, in both men and women, may likely be due to higher serum concentrations of testosterone (Clark et al., 2018; Handelsman et al., 2018).

In this study we investigated the alterations of cortisol and testosterone in female football referees over a complete season lasting 10 months. Previously, we have shown an increase in testosterone and cortisol concentrations during the season of young footballers (Muscella et al., 2019), and male football referees (Muscella et al., 2021), in agreement with data reported for other team sports. Here, we show a cortisol and testosterone rise in the first section of the season meaning the high intensity of physical exercise required to begin the regular season. Afterwards, a slight decrement in hormone circulating concentrations is noted at the end of the season. Regarding any discrepancies in cortisol rebounds as a function of sex, the data remain ineffectual and with conflicting results (Filaire et al., 2001; Kivlighan et al., 2005; Li et al., 2015). As remembered before, physical exercise is a stressor, and the quantity of cortisol produced positively relays upon intensity and length of exercise (Galbo, 1981). Acute physical activity takes to cortisol concentration rise, whereas proper continuative exercise harmonizes cortisol concentration increase over time. In female referees, the biggest change in cortisol occurred mid‐season with its levels increasing on average by about 13.1 µg/dl; this increase is about double that observed in male referees, with starting values, in T0, practically the same (Muscella et al., 2021). Other researchers (Martínez et al., 2010; Muscella et al., 2019) have reported the increased concentration of cortisol in the mid‐season in team sports. When athletes practice a right planned exercise program, cortisol generated after each exercise session is eliminated from the body by 24 h. Hence, the cortisol concentration variations may be joined to stress gathered over the season (Engelmann et al., 2004). Apparently, the referees experimented a time point over the season with high stress, namely at the middle of the season, when the matches, the psychological demand, the training load and the impracticability of stadiums (the mid‐season corresponding to mid‐winter) tired out the footballers. Furthermore, at the end of the season these factors disappeared, then the hormone levels decreased. As mentioned above, the maximum increase in concentration is greater in women (85%) than in men (37%), indicating that women fit differently than men to suchlike physical stress. Presently nothing is known concerning eventual mechanisms taking to the noted sex differences. It is feasible that female athletes are more prone to increased intensity of physical activity through the competition periods than males. It should be remembered that different studies indicate that moderate elevations in the cortisol help athletes in their performances (Stansbury & Gunnar, 1994). In fact, it positively concerns learning, memory and emotions significant to manage their rendering. Conversely, too high increases in cortisol can reduce performance because they interfere with several cognitive tasks (Erickson et al., 2003) and can repress the production of testosterone (Cummings, 1983). The higher levels of cortisol in the T2 period, however, do not suppress those of testosterone which remain higher than the values observed at the beginning of training, in the T0 period.

In addition to cortisol, after the intense period of training, the testosterone concentration of the female referees increased by 3 times in T1 and still much below the male range (in males such increase was 1.5 times, (Muscella et al., 2021) then progressively decreasing in T2 and T3. These increases are the result of good management of workloads and rest periods, as higher levels of this hormone are beneficial during long seasons of intense competition. For its potent anabolic effect on muscle tissue and for promoting competitive behavior, testosterone is considered beneficial for athletic performance (Handelsman et al., 2018; Notelovitz, 2002). Increased testosterone levels significantly improve athletic performance in male (Bahrke & Yesalis, 2004) but there is very poor evidence on the effects of testosterone in women. Furthermore, the issue is highly controversial because there are several rulings in professional sport, although not many investigations have shown connections between testosterone concentrations and strength and muscle mass in female athletes (Bermon & Garnier, 2017; Bermon et al., 2018; Cardinale & Stone, 2006; Eklund et al., 2017). Above all, there is indirect evidence that testosterone in female athletes improves athletic performance. For example, before puberty, when circulating testosterone concentrations are similar in young men and women, there is no gender difference in athletic performance, but after puberty, as the testes produce more testosterone, a large gender difference in circulating testosterone concentrations was measured (Handelsman et al., 2018); hence, an obvious sex difference arises in athletic performance. However, recent results by Hirschberg et al. (Hirschberg et al., 2020), have shown an effect of testosterone on physical performances in women. Studies on elite female athletes also confirm such results. Research has shown that there is greater efficiency in some (pole vault, 800 m running, 400 m running, 400 m hurdles, hammer throw) but not all athletic occurrences related to significantly greater endogenous plasma testosterone in female athletes (Bermon & Garnier, 2017). Furthermore, women with rare congenital conditions (i.e., differences in sexual development), who have higher testosterone production, develop greater lean mass, and improve their physical performance (Handelsman et al., 2018). Accordingly, we here confirm that testosterone is associated with improved performance in female athletes as we have shown a significant association between testosterone concentration and improvement in running time and VO_2max_. These results extend other studies (Bermon et al., 2018) reporting that high testosterone is related to better performance in women middle‐distance running mainly based on the aerobic energy pathway. Bhasin et al. (2003) reported that testosterone increases muscle mass and decreases body fat; we here also found that testosterone significantly decreases body fat percentage during football season. It might be believed that hormone modification through menstrual cycle may concern cortisol and testosterone response to exercise, but various studies show no significant effects either during resistance training (Elliott et al., 2003) or during aerobic training (Haff et al., 2008; Kanaley et al., 1992). Thus, despite significantly lower testosterone levels in women, comparing these results with those obtained on male referees (Muscella et al., 2021) we can assert that there was no significant difference in exercise response between genders. The results suggest, considering the increases in testosterone during the periods evaluated, that it may have a significant part in the anabolic reaction to exercise also in female football referees.

Cortisol and testosterone have an important part in carbohydrate and protein metabolism, acting as competitive agonists at receptor level of muscle cells. Hence, beside separately monitoring cortisol and testosterone, controlling their relative blood concentrations (T/C ratio) over a training season may supply a pertinent clue of anabolic‐catabolic balance, particularly in male athletes (Urhausen et al., 1995). The T/C ratio exiting a significative decrement after workout intensity and duration (Hayes et al., 2010; Meeusen et al., 2013; Urhausen et al., 1995) represents a helpful means in the early detection of overtraining syndrome. However, T/C is less generally and uncontroversial studied in female athletes since female have less testosterone than males (Wood & Stanton, 2012), making the response difficult to interpret. In a 14‐week study in female swimmers reported that the T/C did not change (Santhiago et al., 2011); instead in elite women volleyball players T/C decreased by 30% across measures and then returns to baseline levels, over their competitive season (Roli et al., 2018). Naturally, additional research is needed in this area from a large variety of women's sports for the purpose of build a hormone reference values. In our current study the T/C exhibited little change during season to its completion, with a significant increase in T1 (and at the end of the season for men) (Muscella et al., 2021) suggesting an adaptation at the beginning for women and at the end of the regular sportive year, for men. We can therefore assume that female referees have not been overtrained and respond adequately to training. Cortisol and testosterone blood concentrations and their ratio are a sign of the functional condition of an athlete and should be regularly assayed during the football season in order that coaches and medical team can identify the referees’ training as required. In fact, it is important to observe these changes because they can help maximize referees’ performance and limit any cases of overtraining.

Football refereeing is an intermittent exercise at high intensity, especially of the aerobic type, but also with an effective anaerobic metabolism involvement (Castagna et al., 2007). Thus, for a valid refereeing performance, it is indicated a well‐developed aerobic and anaerobic fitness to cope with match demands in football referees to successfully officiate (Weston et al., 2012). Regarding the aerobic fitness, despite the interest, only few studies reported information on this relevant variable in female referees (Castagna et al., 2007; Weston et al., 2012). In general, the VO_2max_ level observed in female resulted inferior to their male counterparts (Shephard, 2000). Interestingly, here in T3, 64% of female referees achieved the level of male VO_2max_. and all referees improved their aerobic and anaerobic performance, as demonstrated by the increment of YYIRT1 during the football season. As a matter of fact, YYIRT1 is associated to referees’ performance (Krustrup & Bangsbo, 2001), because during this test the anaerobic system is strongly burdened, with the aerobic load next to the maximum values (Muscella et al., 2019). Besides to aerobic fitness, persistent sprint performance is a further critical component for athletes (Girard et al., 2011; Ruscello et al., 2013). The fastest 40‐m time sprint test possessed proper construct potency for the physical evaluation of football referees (Weston et al., 2009). Our results demonstrated that the performance enhanced, and it was also correlated to testosterone variations. Actually, training sessions to which football referees were subjected gave good levels of anaerobic and aerobic fitness. Therefore, female referees holding the necessary abilities are potentially suitable to officiate male matches that are presumed to demand superior external burdens than female competitions.

The change in testosterone and cortisol concentrations heavily related to fitness performances (VO_2max_); accordingly, such variations may be regarded as an endocrine marker of physical fitness in female football referees.

## 5 CONCLUSION

Results demonstrated that the training sessions to which the football referees were subjected have given results of good levels of anaerobic and aerobic fitness and that female referees are eligible to officiate male matches. In addition, training elicites characteristic alterations in endocrine function, in order to preserve body homeostasis in women referees. Coaches and sport researchers must regularly distinguish the hormones variations for gaining athletes’ performance. Lastly, ulterior studies incorporating a bigger population of female football referees are required to endorse these results.

## CONFLICT OF INTEREST

The authors declare that the research was conducted in the absence of any commercial or financial relationships that could be construed as a potential conflict of interest.

## ETHICS STATEMENT

The authors have ethics approval and consent to participate.

## AUTHOR CONTRIBUTIONS

AM supervised the study AM, AB, OS, and S.M performed hypothesis, generation, contributed to the design, data analysis, interpretation of results, and manuscript preparation. GM, OS, and DZ conducted the experiments, tests, and data analysis. AM writing—original draft preparation; SM writing—review and editing. All authors edited and approved the final manuscript.
